# Incidence and predictors of sudden cardiac death in arrhythmogenic right ventricular cardiomyopathy: a pooled analysis^[Author-notes euac014-FM1]^

**DOI:** 10.1093/europace/euac014

**Published:** 2022-03-17

**Authors:** Thomas A Agbaedeng, Kirsty A Roberts, Liam Colley, Jean Jacques Noubiap, David Oxborough

**Affiliations:** Wellcome Centre for Human Genetics, Nuffield Department of Medicine, University of Oxford, Oxford, UK; Adelaide Medical School, Faculty of Health and Medical Sciences, The University of Adelaide, Adelaide, Australia; Centre for Heart Rhythm Disorders, Faculty of Health and Medical Sciences, The University of Adelaide, Adelaide, Australia; Research Institute for Sport and Exercise Sciences, Liverpool John Moores University, Liverpool, UK; HMGBiotech srl, Milan, Italy; School of Medicine and Surgery, The University of Milano-Bicocca, Milano, Italy; Centre for Heart Rhythm Disorders, Faculty of Health and Medical Sciences, The University of Adelaide, Adelaide, Australia; Research Institute for Sport and Exercise Sciences, Liverpool John Moores University, Liverpool, UK

**Keywords:** Risk prediction, Risk stratification, Meta-analysis, Sudden cardiac death, Arrhythmogenic right ventricular cardiomyopathy, International task force criteria, Implantable cardioverter-defibrillator, Ventricular tachycardia, Ventricular fibrillation

## Abstract

**Aims:**

Arrhythmogenic right ventricular cardiomyopathy (ARVC), an inherited heart muscle abnormality, is a major cause of sudden cardiac death (SCD). However, the burden of SCD and risk factors in ARVC are not clearly described. Thus, we estimated the rates and predictors of SCD in ARVC in a meta-analysis.

**Methods and results:**

PubMed, Embase, and Web of Science were searched through 7 April 2021. Prospective studies reporting SCD from ARVC cohorts were included. Data were independently extracted by two reviewers and pooled in a random-effects meta-analysis. Fifty-two studies (*n* = 5485 patients) with moderate-to-low risk of bias were included. The pooled annualized rates of SCD were 0.65 per 1000 [95% confidence interval 0.00–6.43, *I*^2^ 0.00%] in those with an implantable cardioverter-defibrillator (ICD) and 7.21 (2.38–13.79, *I*^2^ 0.0%) in non-ICD cohorts: 7.14 in probands and 8.44 for 2010 Task Force Criteria (TFC). Multivariable predictors of life-threatening arrhythmic events including SCD were: age at presentation [adjusted hazard ratio 0.98 (0.97–0.99)], male sex [2.08 (1.29–3.36)], right ventricular (RV) dysfunction [6.99 (2.17–22.49)], QRS fragmentation [6.55 (3.33–12.90)], T-wave inversion [1.12 (1.02–1.24)], syncope at presentation [2.83 (2.40–4.08)], previous non-sustained ventricular tachyarrhythmia [2.53 (1.44–4.45)], and the TFC score [1.96 (1.02–3.76)], (*P* < 0.05). Predictors of appropriate ICD therapy were RV dysfunction, syncope, and inducible ventricular arrhythmia (*P* < 0.01).

**Conclusion:**

This meta-analysis demonstrates a high burden of SCD in ARVC patients, especially among probands and ARVC defined by the modified TFC. Better strategies are required to improve patient management and prevent SCD in ARVC. PROSPERO ID: CRD42020211761.

## Graphical Abstract

**Graphical Abstract:**
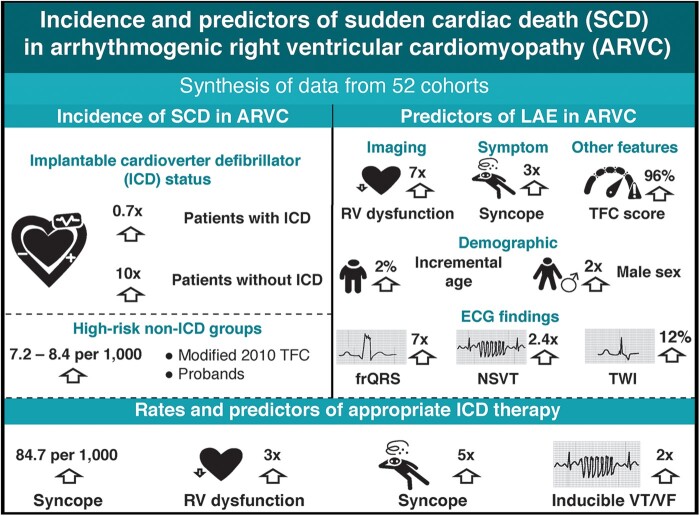

What’s new?

- Herein, we comprehensively estimated the rates and predictors of sudden cardiac death (SCD) in arrhythmogenic right ventricular cardiomyopathy (ARVC) using a systematic review with meta-analysis of 52 cohorts.
- We demonstrate that SCD occurs frequently in ARVC patients, especially among probands and ARVC defined by the modified Task Force Criteria.
- We further show that, in addition to previously identified right ventricular dysfunction and syncope, younger age, QRS fragmentation, and non-sustained ventricular tachycardia are predictors of life-threatening arrhythmic events, including SCD.
- Our findings highlight the need for prospective evaluations of accurate and novel prognostic factors ARVC risk stratification.

## Introduction

The clinical phenotype of arrhythmogenic right ventricular cardiomyopathy (ARVC) encompasses a group of disorders characterized by ventricular arrhythmia and dysfunction, formerly and frequently referred to as ARVC or dysplasia. More recently, however, wider recognition of left-sided dominance and biventricular disease has led to the evolution of the more inclusive term of arrhythmogenic cardiomyopathy.^1,2^

The ARVC is largely a genetic disease that involves intra-myocardial fibro-fatty infiltration, together with atrophy of the ventricular myocardium,^3^ leading to electrical instability and subsequent arrhythmia. With an estimated prevalence of 1:2000 to 1:5000 and a clinical presentation that can vary significantly,^4^ establishing a diagnosis of ARVC is often not straightforward, despite modified Task Force Criteria (TFC).^5^ This is compounded by the fact that individuals with ARVC are at increased risk of sudden cardiac death (SCD), most notably young and athletic populations.^6–8^ Importantly, acute cardiac symptoms, such as arrhythmia and syncope, or SCD can often be the first presentation of the disease in a previously asymptomatic individual.^3,9^ Despite physical exertion being known to influence SCD,^7^ studies have observed that many deaths have occurred at rest or during sleep.^3,10^

Furthermore, the true burden of SCD in ARVC remains unresolved. Current estimates have suggested variable rates for SCD in ARVC patients.^11–13^ However, these results were largely drawn from retrospective cohorts or registries, which are often plagued with poorly ascertained ARVC and SCD.^14^ Hence, the purpose of this systematic review and meta-analysis was to comprehensively summarize available data on the incidence and predictors of SCD in ARVC.

## Methods

### Registration

The current review and meta-analysis protocol was registered with PROSPERO (ID CRD42020211761) and followed the Preferred Reporting Items for Systematic Review and Meta-analyses (PRISMA) guidelines.^15^

### Search strategy

PubMed (MEDLINE), Excerpta Medica Database (Embase), and Web of Science Core Collection were searched to identify studies that reported on the incidence/prevalence, and risk factors of SCD in patients with ARVC, published until 7 April 2021, with no language restriction. The search strategy utilized a number of relevant key terms related to ‘Arrhythmogenic Right Ventricular Cardiomyopathy’ and ‘Sudden Cardiac Death’, including their bibliographic synonyms (Supplementary material online, *Methods S1*). Moreover, the reference lists of relevant articles and reviews were also screened as an additional data source.

### Eligibility criteria and study inclusion

Two investigators (K.A.R. and L.C.) independently screened for eligibility of the records from bibliographic searches based upon their titles and abstracts. At this stage, references of grey literature, including case studies, reports, series, and editorials, were excluded. Cohort and cross-sectional studies were included that reported on the incidence/prevalence of SCD in ARVC, whereas studies were excluded if they did not report any relevant primary data.

Diagnosis of ARVC could be left, right, or biventricular in nature, and the pedigree could be probands, familial, or both. A clinical diagnosis had to have been made using either the 1994, or modified 2010 TFC of ARVC/dysplasia. Studies were included with individuals of any age. However, if studies included patients that were pregnant, had end-stage kidney disease, and/or had terminal cancer, they were excluded.

Full texts of studies with titles and abstracts fulfilling eligibility criteria were subsequently retrieved and screened by the same two independent researchers for eligibility, and included or excluded thereafter. Should studies report data from the same cohort of patients, the study with the largest sample size was included. Any queries or discrepancies in the selection of studies were resolved with consensus or discussed with a third reviewer (T.A.A.).

### Data extraction

Data from eligible studies were extracted by two investigators (K.A.R. and L.C.) using a preconceived data extraction form. The following data were extracted: first author name, year of publication, recruitment period, country of study origin, study design, source of study cohort (and source of control cohort, if any), selection criteria for participant inclusion, follow-up duration, ARVC diagnosis, electrocardiographic(ECG)/Holter parameters, imaging parameters (echocardiographic, magnetic resonance imaging [MRI] findings), information on clinical history, study endpoint (e.g., SCD), study demographic characteristics (mean age and proportion of males), and effect size estimates, such as mean plus/minus standard deviation (±SD), relative risk, and 95% confidence intervals (CI), and hazard ratio (HR) and 95% CI. Any queries (e.g., relevance of data) or discrepancies during the extraction process were either resolved with consensus or discussed with a third investigator (T.A.A.).

Multivariable adjusted effect size estimates were derived from primary analysis that included a composite of life-threatening arrhythmic events (LAE). Additionally, fully adjusted risk estimates for appropriate implantable cardioverter-defibrillator (ICD) shocks were obtained from available studies. For the definitions of outcomes and predictors, refer Supplementary material online, *Methods S2*.

### Risk of bias assessment

The methodological quality of included studies were assessed by two investigators (K.A.R. and L.C.) using the Newcastle Ottawa Quality Assessment Scale (NOS) for assessing Risk of Bias (RoB) in cohort studies that includes three domains: adequacy of selection, comparability of the study group, and ascertainment of outcome.^16^ Studies could get a maximum of nine points: ≤4 = high RoB (poor quality), 5–7 = moderate RoB (moderate quality), 8–9 points = low RoB (good quality).

### Statistical analysis

We performed random-effects meta-analysis of incidence rates of ARVC in SCD and SCD in ARVC using the inverse variance model. We adjusted for variable follow-up durations of the studies to provide annualized event rates. Next, we performed univariable random-effects meta-analyses to assess the association between baseline clinical variables and SCD rates in ARVC, summarizing this as OR (95% CI). We then performed multivariable random-effects meta-analyses using the most adjusted risk estimates associating baseline clinical variables and LAE outcomes, reporting the results as adjusted HR (aHR) and 95% CI. Heterogeneity was assessed by the χ^2^ test on Cochrane’s Q statistic and quantified by *I*^2^ values, assuming that *I*^2^ values of <25%, 50–75%, and >75%, respectively, represent low, moderate, and high heterogeneity.^17^ We assessed small-study effect by funnel plots and tests of funnel plot asymmetry (Egger’s linear regression test), with correction for bias performed using Trim-and-Fill methods. All statistical tests were two-tailed, with significance defined as *P*-value ≤0.05. All analyses were conducted using R version 4.0.3 (The R Foundation for Statistical Computing, Vienna, Austria).

## Results

### Study selection and characteristics

**Figure 1:**
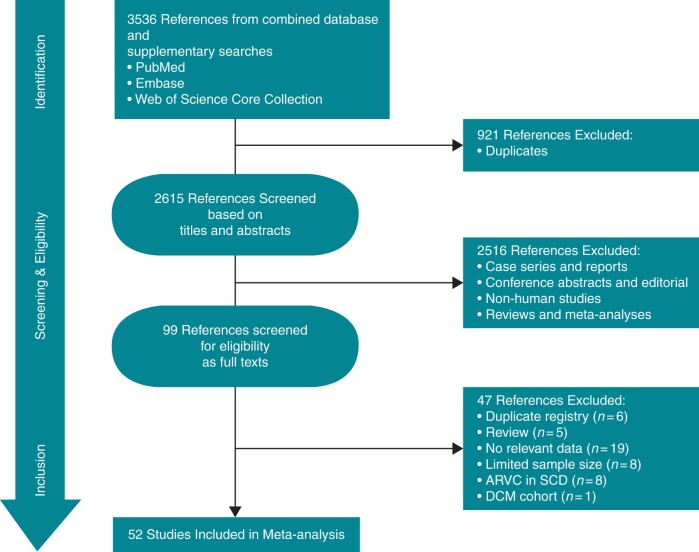
PRISMA flowchart of study selection. ARVC, arrhythmogenic right ventricular cardiomyopathy; DCM, dilated cardiomyopathy; SCD, sudden cardiac death.

Database and supplementary searches retrieved 3536 records, from which 52 articles were finally included (*Figure 1*). The list of included studies and their characteristics are presented in Supplementary material online, *Tables S1–S3*. The included studies reported data from a pooled sample of 5485 patients, were conducted between 1970 and 2011, and published between 1988 and 2021. Most studies were conducted in Europe (61.54%, *n* = 32), were hospital-based (84.62%, *n* = 44), and almost half prospectively reported SCD (63.46%, *n* = 33). Most studies had low (23.08%, *n* = 12) or moderate (65.38%, *n* = 34) RoB (Supplementary material online, *Table S4*).

### Sudden cardiac death incidence in arrhythmogenic right ventricular cardiomyopathy

**Figure 2:**
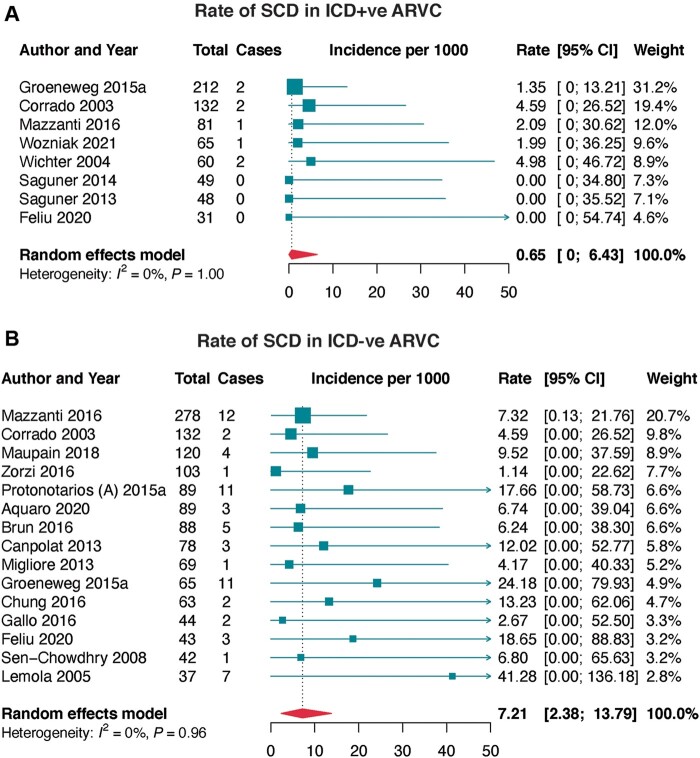
Incidence of sudden cardiac death (SCD) in arrhythmogenic right ventricular cardiomyopathy (ARVC). (*A*) incidence of SCD in ARVC patients with implantable cardioverter-defibrillator (ICD), and (*B*) incidence of SCD in ARVC patients without ICD. CI, confidence interval; ICD−ve, implantable cardioverter-defibrillator negative; ICD+ve, implantable cardioverter-defibrillator positive; SCD, sudden cardiac death.

Eight studies prospectively reported SCD in ARVC patients implanted with ICD (*Table 1* and *Figure 2A*). The pooled annualized incidence rate of SCD was 0.56 per 1000.

**Table 1:** Summary prevalence rates of prospectively reported SCD in arrhythmogenic right ventricular cardiomyopathy patients

Subgroup	Studies (*k*)	Participants		Incidence rate (95% CI)	Heterogeneity		*Egger’s* test (*P*-value)
		No	Events		*I* ^2^	*P*-value	
SCD in ICD positive cohorts							
– Overall	8	678	8	0.65 (0.00–6.43)	0.0%	0.998	0.162
SCD in ICD negative cohorts							
– Overall	15	1340	68	7.21 (2.38–13.79)	0.0%	0.962	0.011
– Probands only	4	354	19	7.14 (0.07–20.99)	0.0%	0.697	0.293
– Probands plus familial	9	901	45	7.24 (1.65–15.41)	0.0%	0.843	0.070
– 2010 TFC	9	890	55	8.44 (2.38–16.99)	0.0%	0.743	0.025
– 1994 and 2010 TFC	2	113	3	3.27 (0.00–28.99)	0.0%	0.962	ND
– European cohorts	12	1080	53	6.65 (1.61–13.93)	0.0%	0.949	0.040
– Trans-Atlantic cohorts	2	197	13	10.02 (0.00–36.79)	0.0%	0.244	ND
Aborted SCD in ICD negative cohorts							
– Overall	8	626	21	4.87 (0.09–13.99)	0.0%	0.667	0.215
– 2010 TFC	4	335	15	7.80 (0.03–23.55)	9.9%	0.344	0.491
– Definite ARVC	2	83	2	3.13 (0.00–35.93)	0.0%	0.998	ND
Aborted SCD and SCD in ICD negative cohorts							
– Overall	7	572	50	12.66 (3.71–25.26)	0.0%	0.622	0.421
– 2010 TFC	3	281	29	15.24 (1.25–39.54)	30.4%	0.238	0.213
– Definite ARVC	2	83	14	20.72 (0.00–68.59)	0.0%	0.361	ND

ARVC, arrhythmogenic right ventricular cardiomyopathy; CI, confidence interval; ICD, implantable cardioverter-defibrillator; ND, not determined; SCD, sudden cardiac death; TFC, Task Force Criteria.

Prospectively reported SCD in patients not on ICD therapy was available in 15 studies (*Table 1* and *Figure 2B*). The overall pooled incidence rate was 7.21 per 1000, with 7.14 per 1000 in cohorts that only enrolled probands and 7.24 per 1000 in those that enrolled both probands and familial ARVC; the difference was not significant (*P* = 0.926). According to the diagnostic criteria for ARVC ascertainment, SCD rate was 8.44 per 1000 on 2010 TFC and 3.27 per 1000 on both 1994 and 2010 TFC (*P* = 0.742).

**Figure 3:**
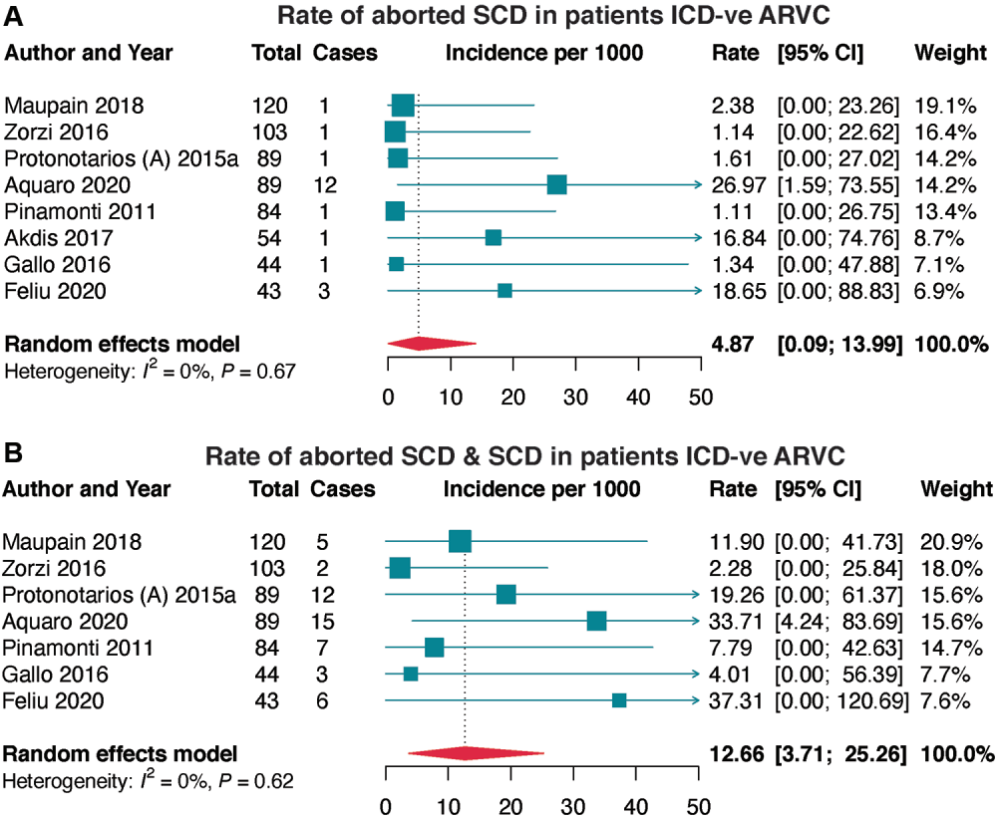
Incidence rates of aborted SCD and both aborted SCD/SCD in ICD−ve ARVC. (*A*) incidence of aborted SCD in ARVC patients without ICD, and (*B*) incidence of both aborted SCD and SCD in ARVC patients without ICD. ARVC, arrhythmogenic right ventricular cardiomyopathy; ICD−ve, implantable cardioverter-defibrillator negative; SCD, sudden cardiac death.

Aborted SCD was prospectively reported in eight cohorts and in patients not on ICD therapy (*Table 1* and *Figure 3A*). The pooled incidence rate of aborted SCD was 4.87 per 1000; 7.80 per 1000 in the ARVC population diagnosed based on the modified 2010 TFC and 3.13 per 1000 in those diagnosed with definite ARVC. Furthermore, for both aborted SCD and SCD (*Figure 3B*), the pooled incidence rate of both aborted SCD and SCD was 12.66 per 1000, from seven prospective studies. This was higher when ARVC was diagnosed by 2010 TFC at 15.24 per 1000, and much higher in the definite ARVC population at 20.72 per 1000.

No evidence of statistical heterogeneity was detected in the main analyses. Additionally, publication bias or small-study effect was not seen in the main pooled analyses (funnel plot asymmetry: *P* > 0.05, *Table 1*), except for SCD in ARVC not on ICD (Supplementary material online, *Figure S1*). After correction for bias, the rate of SCD in ARVC not on ICD was 3.57 per 1000 (Supplementary material online, *Figures S2* and *S3*).

Retrospectively reported SCD, aborted SCD, and both aborted SCD and SCD are summarized as pooled proportions (Supplementary material online, *Figure S5*).

### Predictors of Sudden cardiac death and arrhythmic events in arrhythmogenic right ventricular cardiomyopathy

Univariable analysis of factors associated with SCD was performed using data from 17 studies (Supplementary material online, *Figure S5*). In prospectively reported SCD, three factors were identified, with no significant association with SCD (*P* > 0.05): male sex [OR (95% CI) 1.32 (0.47–3.74)], positive mutation [1.40 (0.05–40.00)], and ICD implant [0.11 (0.01–1.80)]. Similarly, no significant association was noted for five factors identified for retrospectively reported SCD (*P* > 0.05), including age at presentation, male sex, ICD implant, symptomatic status at presentation, asymptomatic status at presentation, or left ventricular (LV) dysfunction.

**Figure 4:**
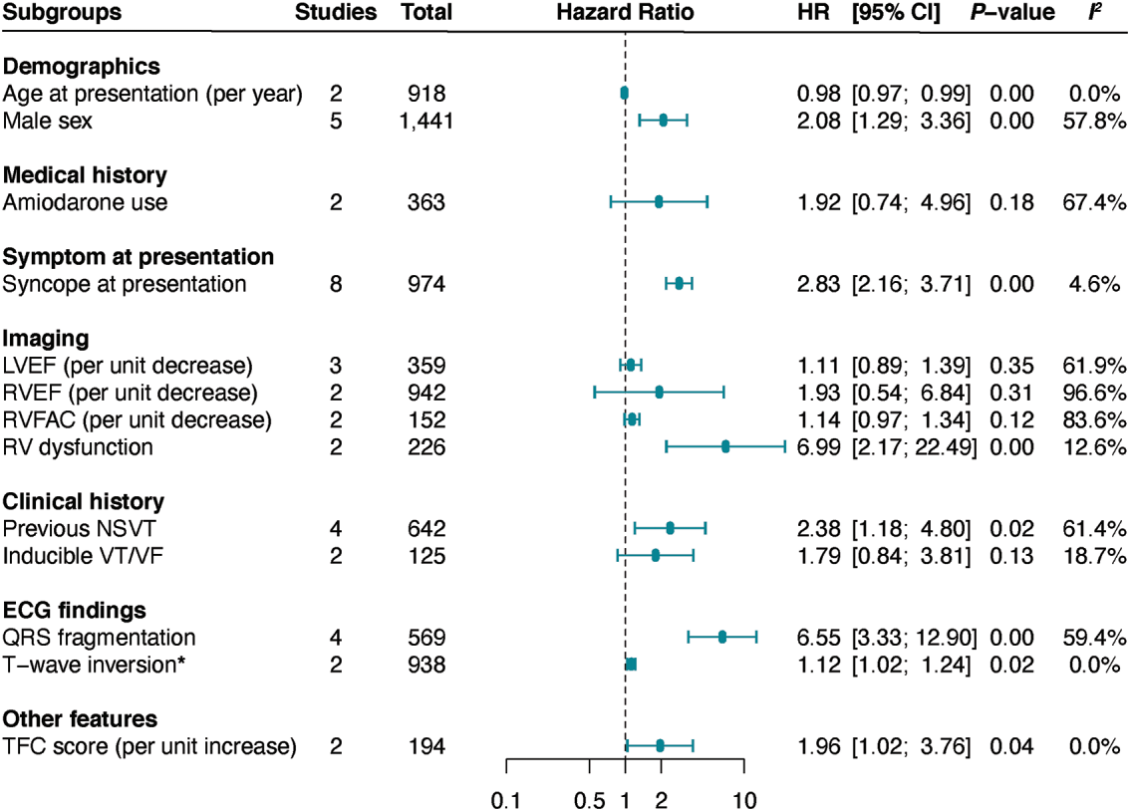
Predictors of life-threatening arrhythmic events in ARVC. Life-threatening arrhythmic events were reported as a composite of SCD, aborted SCD, ventricular tachycardia, ventricular fibrillation, appropriate ICD therapy, or cardiovascular death. HR, hazard ratio; ICD, implantable cardioverter-defibrillator; LVEF, left ventricular ejection fraction; NSVT, non-sustained ventricular tachycardia; RV, right ventricular.

We performed a meta-analysis of predictors of LAEs (including SCD) from multivariable analysis in 19 studies (*Figure 4*). In the pooled analysis, mean age {per yearly increment [aHR (95% CI) 0.98 (0.97–0.99)]}, male sex [2.08 (1.29–3.36)], right ventricular (RV) dysfunction [6.99 (2.17–22.49)], QRS fragmentation [6.55 (3.33–12.90)], T-wave inversion [TWI, 1.12 (1.02–1.24)], syncope at presentation [2.83 (2.40–4.08)], previous history of non-sustained ventricular tachyarrhythmia (VT), non-sustained ventricular tachycardia (NSVT), [2.53 (1.44–4.45)], and the TFC score [1.96 (1.02–3.76)] were predictors of LAEs (*P* < 0.05, *Figure 4*). In contrast, amiodarone therapy, left ventricular ejection fraction (LVEF), RV ejection fraction (RVEF), and inducible VT/ventricular fibrillation (VF) were not significantly predictive of lethal arrhythmic events (*P* > 0.05).

### Meta-analysis of rates and determinants of appropriate implantable cardioverter-defibrillator shocks

**Figure 5:**
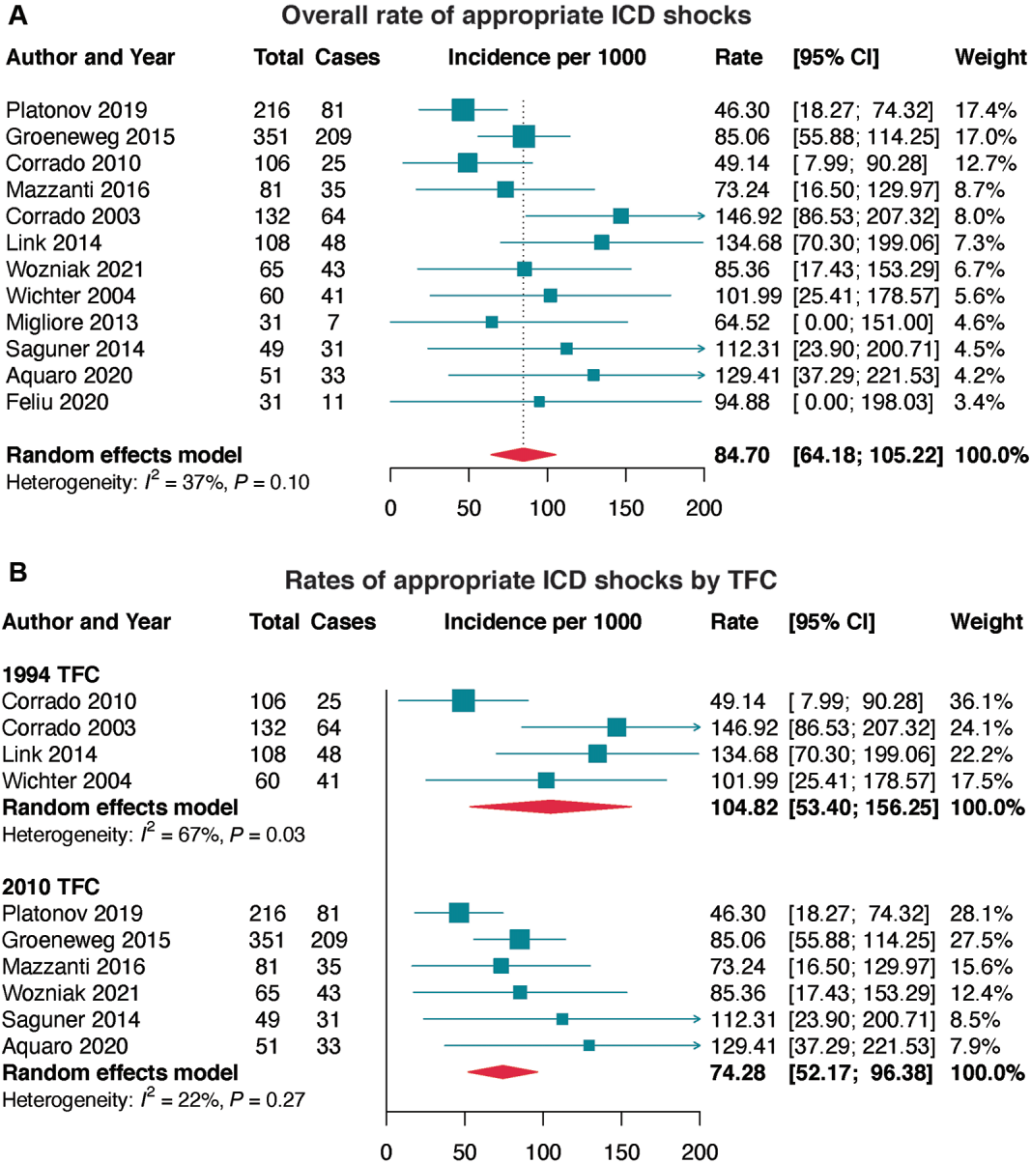
Incidence of appropriate ICD therapy in ARVC. (*A*) Overall incidence rate of appropriate ICD intervention in ARVC, and (*B*) incidence rates of appropriate ICD interventions in ARVC by Task Force Criteria (TFC). ARVC, arrhythmogenic right ventricular cardiomyopathy; ICD, implantable cardiovertr-defibrillator.

Appropriate ICD shocks occurred at an annual rate of 84.70 per 1000 in ARVC, which was highest in definite ARVC, probands, 1994 TFC, and trans-Atlantic cohorts (*Figure 5* and Supplementary material online, *Table S5*).

**Figure 6:**
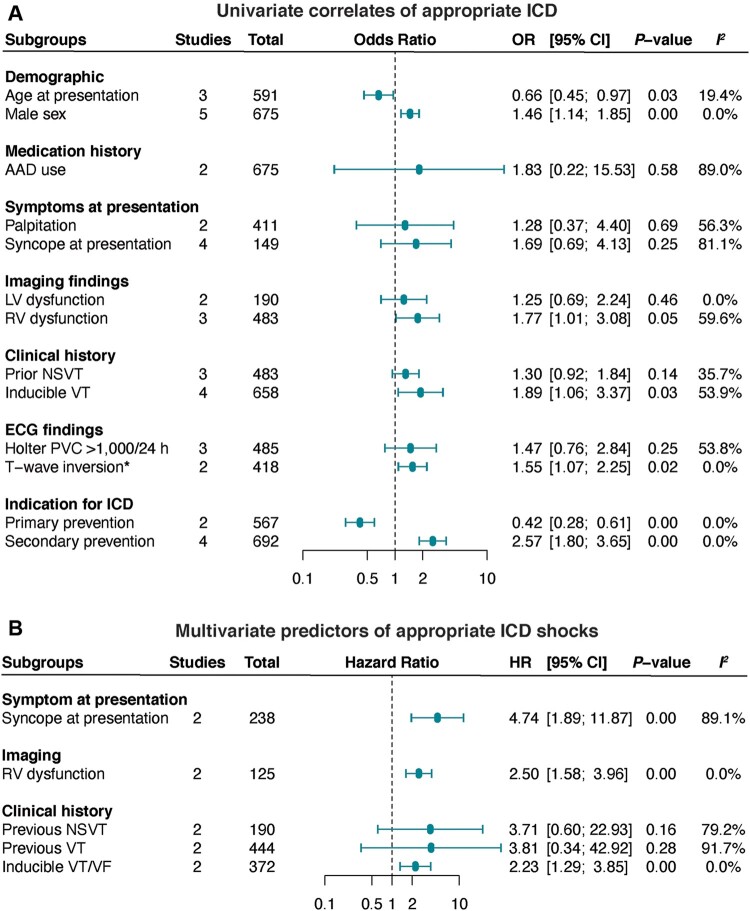
Predictors of appropriate ICD therapies in ARVC. HR, hazard ratio; ICD, implantable cardioverter-defibrillator; LVEF, left ventricular ejection fraction; NSVT, non-sustained ventricular tachycardia; RV, right ventricular.

Univariate correlates of appropriate shocks included: age at presentation (per yearly increment), male sex, RV dysfunction, TWI, inducible VT, and primary (lower risk) and secondary (higher risk) indications for ICD (*P* < 0.05, *Figure 6B*). In the pooled analysis of multivariate estimates, RV dysfunction, syncope at presentation, and inducible VT/VF remained significantly predictive of greater appropriate shocks (*P* < 0.01, *Figure 6B*).

## Discussion

Arrhythmogenic cardiomyopathy is well known to predispose patients to SCD; however, the burden of SCD in ARVC has not been thoroughly evaluated. This meta-analysis of 52 studies demonstrates that (*Graphical Abstract*): (i) the incidence of well-defined SCD from prospective cohorts is low in patients on ICD therapy, but 10× higher in patients not on ICD therapy; (ii) rates of SCD are higher in ARVC diagnosed according to the modified 2010 TFC, probands, and definite diagnosis; and (iii) younger age, RV dysfunction, QRS fragmentation, syncope at presentation, and previous non-sustained VT are multivariate predictors of LAE, including SCD. Furthermore, RV dysfunction, syncope at presentation, and inducible VT/VF are strong predictors of appropriate ICD therapy for SCD.

This meta-analysis shows that the incidence rates of SCD are higher in ARVC patients diagnosed using the modified 2010 TFC, compared with incorporating the original 1994 TFC. The latter was based on structural, functional, ECG, and familial features, which were sorted into major and minor scoring systems for the diagnosis of ARVC.^18^ While the 1994 TFC were able to differentiate between ARVC and related disorders, such as dilated cardiomyopathy or ischaemic heart disease, they have repeatedly shown low sensitivity in detecting early and minor phenotypes, especially among first-degree relatives.^19,20^ Moreover, the original TFC were based predominantly on RV manifestation of the disease and were proposed at a time when clinical presentation of ARVC was heavily dependent upon symptomatic index cases. To improve upon these limitations, the modified criteria were proposed with a view towards improving the diagnostic accuracy of ARVC, thereby improving the specificity among probands and first-degree relatives.^5^ Consequently, application of the 2010 criteria results in a reduced number of patients meeting the ARVC diagnosis, thereby avoiding misdiagnosis and leading to increased capturing of high-risk groups.^21^ Therefore, this confirms that the modified 2010 TFC can improve the risk stratification of ARVC patients.

The goal of the management of ARVC patients is the prevention of SCD. Although several strategies have been used for treating patients, ICD implantation remains the most effective means for disease management and prevention of SCD. A previous meta-analysis of ARVC patients with an ICD demonstrated an annualized cardiac mortality rate of 0.9%.^22^ In patients with definite ARVC, Orgeron *et al*.^23^ reported 2% of the patients having SCD after 8.8 years of follow-up. Consistent with these previous findings, our study shows that the annualized incidence rates of SCD in patients on ICD therapy are as low as 0.56 per 1000. In contrast, our findings demonstrated that a lack of ICD is associated with an almost 10-fold greater incidence of SCD during long-term follow-up. Importantly, SCD incidence rates are highest among probands and those with definite diagnosis of ARVC, with >7% and >12% reported for SCD and both SCD/aborted SCD, respectively.

While SCD prevention is achievable with ICD therapy, accurate identification of at-risk patients is vital for effective disease management. This is particularly important in order to avoid device-related complications. For instance, after 4.8 years of follow-up, Corrado *et al*.^24^ reported inappropriate therapies in 19% of ARVC patients with an ICD, with 17% experiencing device-related complications. Consequently, the latest consensus statement from the International Task Force recommends ICD implantation only in patients with prior aborted SCD or sustained VT with class I recommendation,^25^ while the Heart Rhythm Society recommends it in those with prior cardiac arrest,^26^ sustained VT or syncope, or LVEF ≤35% (with >1 year survival) with class I recommendation. Accordingly, a previous meta-analysis identified male sex, unexplained syncope, prior NSVT and SVT, and inducible VT/VF as univariate correlates of arrhythmia in ARVC.^27^ Consistent with this, using a pooled analysis of fully adjusted models, we show that younger age, male sex, syncope at presentation, RV dysfunction, prior NSVT, QRS fragmentation, TWI, and TFC score are independent predictors of LAE, including SCD, in ARVC. Although these factors are not currently given class I recommendation, they could serve an important role in identifying patients that would most benefit from ICD therapy, while reducing complication rates.

Some of these predictors differ from those reported and included in the prediction model by Cadrin-Tourigny *et al*.^28^ In their study, the investigators found only four variables (male sex, younger age, premature ventricular complex count, and TWI) to be predictive of LAE, but not prior sustained ventricular arrhythmias, RVEF, or LVEF. However, there are some pertinent points that should be noted. First, their prediction model suffers from biases due to the inhomogeneous study population which included both patients with and/or without an ICD and the combined endpoint used for the assessment of the arrhythmic outcome comprised appropriate ICD intervention on VT. Indeed, there is an understanding that appropriate ICD is a poor surrogate of arrhythmic cardiac arrest, given that most VT episodes treated by ICD are self-terminating and haemodynamically stable.^29^ Secondly, since appropriate ICD intervention accounted for the majority of the study outcomes, the model overestimates the true risk of SCD. Thirdly, because only half of total study population had an ICD, the other half of study patients (without an ICD) were prevented from experiencing an appropriate ICD intervention. Thus, the algorithm was based on outcome data that were inhomogeneous and unbalanced in favour of ICD carriers.

Additionally, determining the factors that are predictive of appropriate shocks is very important for informing the effective management of ICD patients. Herein, our results demonstrated that the rates of ICD interventions are highest in definite ARVC and patients with proband status. We identified older age, male sex, RV dysfunction, inducible VT, TWI, and secondary indication as correlates of appropriate therapy. More importantly, our results show that, specifically, syncope at presentation, RV dysfunction, and inducible VT/VF during electrophysiology studies are highly predictive (>2-fold risk) of appropriate ICD therapy. These factors could be used to guide therapy and patient management.

### Limitations

The present findings should be interpreted in light of some limitations. First, the sample sizes of the individual studies were relatively low. Secondly, the multivariable analyses of predictors of arrhythmic events included a composite of SCD, VF, VT, and cardiac death. Indeed, this may have overestimated the associated risks when compared with having only SCD. Future studies should only focus on reporting SCD and/or aborted SCD as sole primary endpoints. Thirdly, due to a lack of reported data, prospectively reported SCD in non-ICD cohorts could not be estimated in select groups, such as endurance exercise, mutation carriers, anti-arrhythmic drugs, or familial disease. Additionally, the burden of SCD in definite ARCV could not be compared with borderline or probable ARVC groups. This was because the included studies did not characterize events by the type of ARVC.

## Conclusion

This meta-analysis demonstrates that the incidence of well-defined SCD from prospective cohorts is very low in patients with ARVC on ICD therapy. However, in patients without an ICD, the incidence rates are almost 10-fold higher, with the highest rates seen in patients diagnosed according to the modified 2010 TFC, probands, and definite diagnosis. Importantly, the present findings demonstrate that, in addition to previously identified RV dysfunction and syncope at presentation, younger age, QRS fragmentation, and previous non-sustained VT are predictors of LAE, including SCD. Further large-scale, prospective studies are warranted to better evaluate accurate and novel prognostic factors, QRS fragmentation and NSVT, for disease risk stratification.

## Supplementary material

Supplementary material is available at *Europace* online.

## Supplementary Material

euac014_Supplementary_Data

## Data Availability

The data that support the findings of this study are available from the corresponding author upon reasonable request.
