# Supplementary material for: Incidence and predictors of sudden cardiac death in arrhythmogenic right ventricular cardiomyopathy: a pooled analysis
Source: Europace. 2022 Mar 17;24(10):1665–74. doi: 10.1093/europace/euac014 (PMC9559905; doi:10.1093/europace/euac014)
Supplement: euac014_Supplementary_Data [file euac014_supplementary_data.docx]

**Incidence and predictors of sudden cardiac death in arrhythmogenic right ventricular cardiomyopathy: evidence from a pooled analysis**

**Authors:** Thomas A. Agbaedeng, PhD;^1,2*^ Kirsty A. Roberts, PhD;^3^ Liam Colley, MSc;^4^ Jean Jacques Noubiap, MD;^1^ David Oxborough, PhD.^3^

**From:** ^1^Centre for Heart Rhythm Disorders, The University of Adelaide, Adelaide, Australia; ^2^Centre for Cardiometabolic Experimentation and Innovation, Adelaide, Australia; ^3^Research Institute for Sport and Exercise Sciences, Liverpool John Moores University, Liverpool, UK; ^4^HMGBiotech srl, Milan, Italy; ^5^School of Medicine and Surgery, The University of Milano-Bicocca, Milano, Italy

***Corresponding author:**

Dr Thomas A. Agbaedeng

Centre for Heart Rhythm Disorders, Level 8 North, SAHMRI,

2 North Terrace, Adelaide, SA 5000. AUSTRALIA

Telephone: +61883139000; E-mail: [thomas.agbaedeng@outlook.com](mailto:thomas.agbaedeng@outlook.com); ORCiD: <https://orcid.org/0000-0002-4399-9139>

Table of Contents

[Supplementary Method S1. Search Strategy 3](#_Toc85342244)

[Supplementary Method S2. Definitions of predictors and outcomes 4](#_Toc85342245)

[Supplementary Tables 5](#_Toc85342246)

[Table S1. Summarized study characteristics 5](#_Toc85342247)

[Table S2. Characteristics of included studies 6](#_Toc85342248)

[Table S3. Baseline clinical characteristics 10](#_Toc85342249)

[Table S4. Newcastle-Ottawa risk of bias estimation 13](#_Toc85342250)

[Table S5. Summary of Annualised Incidence Rates of Appropriate Implantable-Cardioverter Defibrillator Therapy 16](#_Toc85342251)

[Supplementary Figures 17](#_Toc85342252)

[Figure S1. Funnel plot of incidence rate of prospectively reported SCD in ICD-ve ARVC 17](#_Toc85342253)

[Figure S2. Bias-corrected incidence rate of prospectively reported SCD in ICD-ve ARVC 18](#_Toc85342254)

[Figure S3. Funnel plot of bias-corrected incidence of prospectively reported SCD in ICD-ve ARVC 19](#_Toc85342255)

[Figure S4. Pooled prevalence of retrospectively reported SCD or aborted SCD 20](#_Toc85342256)

[Figure S5. Univariate correlates of SCD 21](#_Toc85342257)

[Included Studies 22](#_Toc85342258)

# Supplementary Method S1. Search Strategy

The following keywords were used:

**PubMed:** {(Arrhythmogenic Right Ventricular Dysplasia[mh] OR Arrhythmogenic Right Ventricular Dysplasia[tiab] OR Arrhythmogenic Right Ventricular Cardiomyopathy-Dysplasia[mh] OR Arrhythmogenic Right Ventricular Cardiomyopathy-Dysplasia[tiab] OR Arrhythmogenic Right Ventricular Cardiomyopathy[tiab] OR ARVD-C[tiab] OR ARVD[tiab] OR ARVC[tiab]) AND (Sudden Cardiac Death[mh] OR Sudden Cardiac Death[tiab] OR Sudden Cardiac Arrest[mh] OR Sudden Cardiac Arrest[tiab] OR SCD[tiab] OR SCA[tiab])}

**EMBASE:** {('heart right ventricle dysplasia'/exp OR 'heart right ventricle dysplasia':ti,ab OR ‘Arrhythmogenic right ventricular cardiomyopathy’/exp OR ‘Arrhythmogenic right ventricular cardiomyopathy’:ti,ab OR ‘Arrhythmogenic right ventricular dysplasia’/exp OR ‘Arrhythmogenic right ventricular dysplasia’:ti,ab OR ARVD:ti,ab OR ARVC:ti,ab) AND ('sudden cardiac death'/exp OR 'sudden cardiac death':ti,ab)}

**Web of Science Core Collection:** {(Arrhythmogenic Right Ventricular Dysplasia OR Arrhythmogenic Right Ventricular Cardiomyopathy-Dysplasia OR Arrhythmogenic Right Ventricular Cardiomyopathy OR ARVD-C OR ARVD OR ARVC) AND (Sudden Cardiac Death OR Sudden Cardiac Arrest)

## Supplementary Method S2. Definitions of predictors and outcomes

| **OUTCOME** | **DEFINITION** |
| --- | --- |
| ***Life-threatening arrhythmic events (LAE)*** | Sudden cardiac death (SCD), aborted SCD, ventricular tachycardia [VT] or VT storm, ventricular fibrillation/flutter (VF), and/or appropriate implantable cardioverter-defibrillator (ICD) therapy |
| ***Aborted SCD*** | sudden cardiac arrest, occurring within 1 h following the onset of acute symptoms, which was successfully reversed by any resuscitation manoeuvre (i.e., defibrillation or cardiopulmonary resuscitation) |
| ***Appropriate ICD therapy*** | ICD intervention (i.e., shocks or anti-tachycardia overdrive pacing) triggered in response to ventricular tachyarrhythmia (VT/VF) and documented in intracardiac electrocardiographic (ECG) data |
| ***Definite arrhythmogenic right ventricular cardiomyopathy (ARVC)*** | ARVC diagnosis made through family screening |
| ***Familial ARVC*** | ARVC diagnosis made through family screening |
| ***Fragmented QRS*** | Deflections at the beginning of the QRS complex, either top of the R-wave or nadir of the S-wave, on ≥2 precordial leads |
| ***Inducible VT/VF*** | Sustained ventricular tachyarrhythmia (VF or VT [of ≥30 s in duration or requiring cardioversion due to haemodynamic compromise]) induced during electrophysiology study |
| ***Non-sustained VT*** | ≥3 consecutive ventricular beats at a rate of >100 beats per minute or lasting <30 seconds in duration and without haemodynamic compromise |
| ***Proband*** | An individual in whom the diagnosis of ARVC was confirmed in a family, which was independent of family screening |
| ***Right ventricular (RV) dysfunction*** | Fractional area change ≤40%, tricuspid plane annulus systolic excursion <17 mm on a transthoracic echocardiography (TTE) in 4-chamber view, or RV ejection fraction (RVEF) <40% by cardiac MRI |
| ***SCD*** | Death of cardiac origin that occurred unexpectedly within 1 h of the onset of new symptoms or a death that was unwitnessed and unexpected. |
| ***Syncope*** | A sudden and transient loss of consciousness with spontaneous (i.e., not requiring electrical cardioversion) recovery |
| ***Sustained VT*** | VT lasting ≥30 s or <30 but requiring electrical or pharmacological cardioversion |
| ***T-wave inversion*** | Inverted T waves in precordial leads (V1–V3) |
| ***TFC score*** | 5-year ARVC risk score based on the number of TFC for ICD implantation, including: NSVT, cardiogenic syncope, and moderate-to-severe LV or RV dysfunction |

# Supplementary Tables

## Table S1. Summarized study characteristics

| **Characteristics** | **N = 52** |
| --- | --- |
| Total population | 5,951 |
| Year of publication, range | 1988–2021 |
| Recruitment year of participants, range | 1970–2019 |
| Setting |  |
| - Hospital-based | 44 |
| - Registry | 8 |
| Timing of data collection |  |
| - Prospective | 33 |
| - Retrospective | 15 |
| - Both | 4 |
| Regions |  |
| - Europe | 32 |
| - Northern America | 6 |
| - Trans-Atlantic | 5 |
| - Asia | 8 |
| - Oceania | 1 |
| Sampling |  |
| - Selected | 1 |
| - Consecutive | 37 |
| - Not reported | 14 |
| Risk of bias (Q-Genie) |  |
| - Low | 12 |
| - Moderate | 34 |
| - High | 6 |

## Table S2. Characteristics of included studies

| **Author** | **Year** | **Recruitment period** | **Follow-up (years)** | **Country** | **Region** | **Study name** | **Design** | **Sampling** | **Setting** | **Select criteria** | **Data collection** | **Pedigree** | **Diagnosis** | **ACM definition** | **Patients** |
| --- | --- | --- | --- | --- | --- | --- | --- | --- | --- | --- | --- | --- | --- | --- | --- |
| Akdis | 2017 | NR | 1.1 | Switzerland | Europe | Zurich ARVC Program | Cohort | Consecutive | Hospital | 2010 TFC | Prospective | NR | NR | 2010 TFC | 54 |
| Blusztein | 2018 | 2007-2017 | NR | Australia | Oceania | NR | Cohort | Selected | Hospital | 2010 TFC for ARVC diagnosis (or features of ARVC at postmortem) | Retrospective | Proband | Biventricular | 2010 TFC | 44 |
| Brun | 2016 | NR | 9.1 | Italy/Netherlands | Europe | NIH Study, Familial Cardiomyopathy Registry, Utrecht Registry | Cohort | Consecutive | Hospital | 2010 TFC | Prospective | Proband | Right | 2010 TFC | 88 |
| Cadrin-Tourigny | 2019 | NR | 4.8 | USA/Montreal/Switzerland/Netherlands/Norway/Sweden | Trans-Atlantic | NR | Cohort | Consecutive | Hospital | 2010 TFC | Prospective | Both | NR | 2010 TFC | 528 |
| Canpolat | 2013 | NR | 3.2 | Turkey | Europe | NR | Cohort | NR | Hospital | 2010 TFC | Retrospective and Prospective | Both | Right | 2010 TFC | 78 |
| Cappelletto | 2018 | 1982-2015 | 7.6 | Italy | Europe | Trieste registry | Cohort | Consecutive | Hospital | 2010 TFC | Prospective | Both | Biventricular | 2010 TFC | 98 |
| Chan | 2015 | 2008-2014 | 2.5 | Taiwan | Asia | Taiwan cohort | Cohort | Consecutive | Hospital | 2010 TFC | Prospective | Both | NR | 2010 TFC | 59 |
| Chung | 2013 | 1998-2012 | 1.8 | Taiwan | Asia | Taiwan cohort | Cohort | Consecutive | Hospital | 2010 TFC | Retrospective | Both | NR | 2010 TFC | 88 |
| Chung | 2016 | 2006-2013 | 2.4 | Taiwan | Asia | Taiwan cohort | Cohort | Consecutive | Hospital | 2010 TFC | Prospective | Both | Biventricular | 2010 TFC | 63 |
| Corrado | 2003 | 1992-2001 | 3.3 | Italy/USA | Trans-Atlantic | NR | Cohort | Consecutive | Hospital | 1994 TFC for ARVC diagnosis& ICD implantation with fu 6 months | Prospective | Proband | Biventricular | 1994 TFC | 132 |
| Corrado | 2010 | NR | 4.6 | Italy/Germany/England/USA | Trans-Atlantic | NR | Cohort | Consecutive | Hospital | 1994 TFC | Prospective | Proband | Biventricular | 1994 TFC | 106 |
| Denis | 2014 | 2000-2013 | 6.4 | France | Europe | NR | Cohort | Consecutive | Hospital | 1994 & 2020 TFC | Retro- & prospective | Proband | NR | 2010 TFC | 35 |
| Gallo | 2016 | 1970-2014 | 17 | Italy | Europe | NR | Cohort | NR | Hospital | Patients who fulfilled the 1994 TFC for ARVC diagnosis | Prospective | Both | Right | 1994 TFC & post hoc 2010 TFC | 68 |
| Groeneweg | 2015 | NR | NR | USA, Netherlands | Trans-Atlantic | John Hopkin's ARVD/C Registry, Dutch ARVD/C Registry | Cohort | NR | Registry | Index-patients fulfilling 2010 TFC for ARVD/C | Retrospective and Prospective | Both | Right | 2010 TFC | 646 |
| Hulot | 2004 | 1977-2000 | 8.1 | France | Europe | NR | Cohort | Consecutive | Hospital | 1994 TFC | Retrospective | Proband | Biventricular | 1994 TFC | 130 |
| Incardi | 2014 | NR | NR | Italy | Europe | NR | Cohort | NR | Hospital | 1994 TFC | Retrospective | Both | Biventricular | 1994 TFC | 47 |
| James | 2013 | NR | 8.4 | USA | North America | NR | Cohort | NR | Registry | 2010 TFC | Prospective | Both | NR | 2010 TFC | 87 |
| Kikuchi | 2016 | 1974-2012 | 10.2 | Japan | Asia | NR | Cohort | NR | Hospital | 2010 TFC | Retrospective | Both | Right | 2010 TFC | 90 |
| Kimura | 2018 | 2007-2013 | 3.5 | Japan | Asia | NR | Cohort | Consecutive | Hospital | 2010 TFC | Retrospective | Proband | Right | 2010 TFC | 40 |
| Lemola | 2005 | NR | 4.6 | Switzerland | Europe | NR | Cohort | Consecutive | Hospital | 1994 TFC | Retrospective | Both | Right | 2010 TFC | 61 |
| Li | 2012 | 2000-2010 | 5.7 | Taiwan | Asia | NR | Cohort | Consecutive | Hospital | Modified 2010 TFC | Retrospective | Both | Right | Modified 2010 TFC | 30 |
| Ma | 2009 | 1998-2006 | 3 | China | Asia | NR | Cohort | Consecutive | Hospital | 1994 TFC | Retrospective | Both | Right | 1994 TFC | 39 |
| Maupain | 2018 | 2000-2010 | 3.5 | France | Europe | NR | Cohort | Consecutive | Hospital | Patients with definite or borderline ARVC/D diagnosis who underwent EPS and without ICD implant | Prospective | Both | Right | NR | 137 |
| Mazzanti | 2016 | 1999-2014 | 5.9 | Italy | Europe | NR | Cohort | Consecutive | Hospital | 2010 TFC | Prospective | Both | Biventricular | 2010 TFC | 301 |
| Migliore | 2013 | NR | 3.5 | Italy | Europe | NR | Cohort | Consecutive | Hospital | 1994 TFC & 2010 TFC | Prospective | Proband | Right | 1994 TFC & 2010 TFC | 69 |
| Nava | 1988 | NR | NR | Italy | Europe | NR | Cohort | Consecutive | Hospital | NR | Retrospective | Both | Right | NR | 42 |
| Peters | 1995 | NR | NR | Germany | Europe | NR | Cohort | NR | Hospital | NR | Retrospective | Both | Biventricular | NR | 60 |
| Peters | 2012 | 1986-2008 | 6.3 | Germany | Europe | NR | Cohort | NR | Hospital | 1994 TFC | Retrospective | Both | NR | 1994 TFC | 305 |
| Pinamonti | 2011 | 1976-2008 | 10.7 | Italy | Europe | Trieste registry | Cohort | Consecutive | Hospital | 1994 TFC | Prospective | Both | Right | 1994 TFC | 98 |
| Protonotarios | 2015a | NR | 7 | Greece | Europe | NR | Cohort | Consecutive | Hospital | 2010 TFC | Prospective | Both | Biventricular | 2010 TFC | 105 |
| Protonotarios | 2015b | NR | 9 | Greece | Europe | NR | Cohort | Consecutive | Hospital | 2010 TFC | Prospective | Proband | Biventricular | 2010 TFC | 86 |
| Roguin | 2004 | 2002 | 3.5 | USA | North America | NR | Cohort | Consecutive | Hospital | 1994 TFC | Prospective | Both | Right | 1994 TFC | 42 |
| Saguner | 2013 | NR | 9.8 | Switzerland | Europe | Zurich ARVC Program | Cohort | Consecutive | Hospital | 2010 TFC | Retrospective | Proband | Biventricular | 2010 TFC | 62 |
| Saguner | 2014 | 1987-2013 | 4.6 | Switzerland | Europe | NR | Cohort | NR | Hospital | ACM patients who had 12-lead ECG | Prospective | Both | NR | 2010 TFC | 111 |
| Sen-Chowdhry | 2008 | NR | 3.5 | UK | Europe | NR | Cohort | NR | Hospital | Had family history of ACM, presence of ventricular arrhythmia, T-wave inversion, and LV disease | Prospective | NR | Left | NR | 42 |
| Turrini | 2001 | NR | NR | Italy | Europe | NR | Cohort | Consecutive | Hospital | 1994 TFC | Retrospective | NR | Right | 1994 TFC | 60 |
| Wang | 2018 | 1999- | 7.6 | USA | North America | John Hopkin's ARVD/C Registry | Cohort | Consecutive | Hospital | 2010 TFC | Prospective | Both | Right | 2010 TFC | 131 |
| Wichter | 2004 | 1991-2002 | 6.7 | Germany | Europe | NR | Cohort | Consecutive | Hospital | 1994 TFC | Prospective | NR | Right | 1994 TFC | 60 |
| Zorzi | 2016 | NR | 8.5 | Italy | Europe | NR | Cohort | Consecutive | Hospital | 2010 TFC | Prospective | Both | NR | 2010 TFC | 116 |
| Aquaro | 2020 | NR | 5 | Italy | Europe | PROAC registry | Cohort | Consecutive | Registry | 2010 TFC | Prospective | Both | NR | 2010 TFC | 140 |
| Cadrin-Tourigny | 2021 | NR | 5.75 | Canada, Netherlands, Norway, Sweden, Switzerland, USA | Trans-Atlantic | Multiple registries | Cohort | Consecutive | Registry | 2010 TFC | Prospective | Both | NR | 2010 TFC | 864 |
| Dominguez | 2020 | NR | NR | Spain | Europe | NR | Cross-sectional | NR | Hospital | Family history of SCD | Prospective | Familial | NR | NR | 62 |
| Feliu | 2020 | 2010-2018 | 3.74 | Spain | Europe | NR | Cohort | Consecutive | Hospital | Sen-Chowdhry criteria | Prospective/retrospective | NR | Left | NR | 74 |
| Liang | 2020 | 2000-2019 | 6.08333333 | China | Asia | NR | Cohort | Consecutive | Hospital | 2010 TFC | Retrospective | NR | Right | 2010 TFC | 284 |
| Lie | 2021 | NR | 7.6 | Norway | Europe | NR | Cohort | Consecutive | Hospital | 2010 TFC | Prospective | Both | Left | 2010 TFC | 168 |
| Saguner | 2014 | 2011-2014 | 5.63333333 | Switzerland | Europe | Zurich ARVC Program | Cohort | NR | Hospital | 2010 TFC | Prospective | NR | NR | 2010 TFC | 70 |
| Scheirlynck | 2020 | 2006-2018 | NR | Norway | Europe | NR | Cohort | Consecutive | Hospital | 2010 TFC | Prospective | Both | NR | 2010 TFC | 141 |
| Wozniak | 2021 | 1996-2016 | 7.75 | Poland | Europe | NR | Cohort | Consecutive | Hospital | 2010 TFC | Prospective | Proband | NR | 2010 TFC | 65 |
| Bhonsale | 2011 | 1995- | 4.7 | USA | North America | John Hopkin's ARVD/C Registry | Cohort | NR | Registry | 2010 TFC | Prospective | Both | NR | 2010 TFC | 84 |
| Link | 2014 | NR | 3.3 | USA | North America | NAMS ARVC | Cohort | NR | Registry | NR | Prospective | NR | NR | 1994 TFC | 108 |
| Oregon | 2017 | 1999- | 7.66666667 | USA | North America | John Hopkin's ARVD/C Registry | Cohort | Consecutive | Registry | Definite diagnosis based on 2010 TFC, ICD implantation, and ≥30 days of post-ICD follow-up | Prospective | Both | NR | 2010 TFC | 312 |
| Platonov | 2019 | 2010-2017 | 8.13888889 | Denmark, Norway, and Sweden | Europe | Nordic ARVC Registry | Cohort | Consecutive | Registry | Diagnosis by 2010 TFC | Prospective | Both | NR | 2010 TFC | 216 |

ACM, arrhythmogenic cardiomyopathy; ARVC, arrhythmogenic right ventricular cardiomyopathy; ARVD/C, arrhythmogenic right ventricular dysplasia or cardiomyopathy; ECG, electrocardiograph; NAMS ARVC, North American Multidisciplinary Study of ARVC; NIH, National Institute of Health; NR, not reported; PROAC, Prognostic Value of Magnetic Resonance Phenotype in Patients with Arrhythmogenic Right Ventricular Cardiomyopathy; TFC, International Taskforce Criteria

## Table S3. Baseline clinical characteristics

| **Author** | **Year** | **Mean age (years)** | **Male sex (%)** | **LVD (%)** | **RVD (%)** | **ICD use (%)** | **AAD (%)** | **VT/VF ablation (%)** | **FHx SCD (%)** | **FHx ACM (%)** | **Prior SVT (%)** | **Prior NSVT (%)** | **Positive Mutation (%)** | **Symptomatic (%)** | **Asymptomatic (%)** |
| --- | --- | --- | --- | --- | --- | --- | --- | --- | --- | --- | --- | --- | --- | --- | --- |
| Akdis | 2017 | 51.3 | 72.0 | NR | NR | NR | NR | NR | NR | NR | NR | NR | 24.0 | NR | NR |
| Blusztein | 2018 | NR | 75.0 | NR | NR | NR | NR | NR | NR | NR | NR | NR | NR | NR | NR |
| Brun | 2016 | 38.1 | 68.0 | NR | NR | 0.0 | 76.0 | 3.4 | NR | NR | NR | NR | NR | NR | NR |
| Cadrin-Tourigny | 2019 | 38.2 | 44.7 | NR | NR | 41.3 | 15.5 | NR | NR | NR | NR | 43.8 | 64.4 | 58.1 | 41.9 |
| Canpolat | 2013 | 31.3 | 65.4 | NR | NR | 59.0 | 33.3 | NR | NR | 26.9 | NR | NR | NR | NR | NR |
| Cappelletto | 2018 | 37.0 | 67.0 | 28.0 | 75.0 | 18.0 | NR | NR | 17.0 | 41.0 | NR | 56.0 | NR | NR | NR |
| Chan | 2015 | 47.6 | 59.3 | NR | NR | 42.4 | NR | NR | NR | NR | 78.0 | NR | NR | 100.0 | 0.0 |
| Chung | 2013 | 41.5 | 75.0 | NR | NR | NR | NR | NR | NR | NR | NR | NR | NR | NR | NR |
| Chung | 2016 | 44.7 | 60.3 | NR | NR | 30.2 | 98.4 | NR | 15.9 | NR | 25.4 | NR | 46.0 | NR | NR |
| Corrado | 2003 | 40.0 | 70.0 | NR | NR | 85.0 | 83.0 | NR | 3.0 | 3.0 | 62.0 | 9.0 | NR | NR | NR |
| Corrado | 2010 | 35.6 | 67.0 | 25.0 | 92.0 | 100.0 | 52.0 | NR | 46.0 | NR | NR | 53.0 | NR | 39.0 | NR |
| Denis | 2014 | 41.9 | 65.7 | NR | NR | NR | NR | NR | 8.6 | 22.9 | 31.4 | 23.5 | NR | 77.1 | 22.9 |
| Gallo | 2016 | 31.0 | 69.0 | 26.1 | 45.5 | 35.0 | 40.9 | NR | 32.0 | 26.0 | 22.0 | NR | NR | 79.0 | 21.0 |
| Groeneweg | 2015 | NR | NR | NR | NR | NR | NR | NR | NR | NR | NR | NR | NR | NR | NR |
| Hulot | 2004 | 31.8 | 76.9 | 10.0 | 53.8 | 7.7 | 92.0 | NR | NR | NR | NR | NR | NR | 93.8 | 6.2 |
| Incardi | 2014 | 37.0 | 66.0 | NR | NR | NR | NR | NR | 17.0 | 6.4 | NR | NR | NR | 59.6 | 40.4 |
| James | 2013 | 44.0 | 52.9 | NR | NR | 8.0 | NR | NR | NR | NR | 74.0 | NR | NR | 51.0 | 46.0 |
| Kikuchi | 2016 | 44.0 | 76.0 | NR | 98.0 | 18.0 | 63.0 | 34.0 | NR | 10.0 | 71.0 | 82.0 | NR | NR | NR |
| Kimura | 2018 | 52.5 | 78.0 | NR | NR | 0.0 | NR | 45.0 | NR | NR | 80.0 | NR | NR | NR | NR |
| Lemola | 2005 | 44.0 | 72.0 | NR | NR | NR | NR | NR | NR | 16.4 | NR | NR | NR | 95.1 | 4.9 |
| Li | 2012 | 48.0 | 63.3 | 16.7 | 66.7 | 43.3 | 93.3 | 33.3 | NR | NR | NR | NR | NR | NR | NR |
| Ma | 2009 | NR | 85.0 | 18.0 | 53.0 | NR | NR | NR | 7.7 | NR | NR | NR | NR | NR | NR |
| Maupain | 2018 | 37.0 | 78.0 | 9.0 | 27.0 | 0.0 | 51.0 | NR | 18.0 | NR | 31.0 | 38.0 | NR | 69.0 | 31.0 |
| Mazzanti | 2016 | 38.0 | 58.0 | 1.0 | 9.0 | 26.9 | 39.5 | 9.0 | 26.0 | 64.0 | 13.2 | NR | NR | 9.3 | NR |
| Migliore | 2013 | 36.0 | 68.1 | NR | 100.0 | 44.0 | 82.0 | NR | 23.0 | 17.0 | 13.0 | 63.0 | NR | 32.0 | NR |
| Nava | 1988 | 25.0 | 66.7 | NR | NR | NR | NR | NR | NR | NR | NR | NR | NR | NR | NR |
| Peters | 1995 | 41.4 | 55.0 | NR | NR | NR | NR | NR | 0.0 | NR | 11.7 | 78.3 | NR | NR | NR |
| Peters | 2012 | 46.3 | 49.9 | NR | NR | 13.1 | NR | NR | NR | NR | NR | NR | NR | NR | NR |
| Pinamonti | 2011 | 34.0 | 68.0 | 45.9 | 100.0 | NR | 58.0 | NR | NR | 46.0 | 23.0 | 34.0 | NR | 73.0 | 27.0 |
| Protonotarios | 2015a | NR | 53.0 | 27.0 | 51.0 | 15.0 | 28.0 | NR | NR | NR | NR | NR | 100.0 | NR | NR |
| Protonotarios | 2015b | 40.0 | 59.0 | 31.0 | 76.0 | 36.0 | NR | NR | NR | NR | NR | NR | NR | NR | NR |
| Roguin | 2004 | 36.0 | 52.0 | NR | 50.0 | 100.0 | NR | 21.4 | NR | 28.6 | 4.8 | NR | NR | NR | NR |
| Saguner | 2013 | 42.3 | 68.0 | 24.0 | 48.0 | NR | NR | NR | NR | NR | 71.0 | 23.0 | NR | NR | NR |
| Saguner | 2014 | 43.0 | 64.0 | NR | NR | NR | 36.0 | NR | 10.0 | NR | NR | NR | NR | 32.0 | NR |
| Sen-Chowdhry | 2008 | 44.0 | 52.3 | 100.0 | 86.0 | NR | NR | 66.0 | 43.0 | 64.0 | NR | NR | NR | 71.0 | 29.0 |
| Turrini | 2001 | 24.5 | 85.0 | 93.3 | 31.7 | NR | NR | NR | NR | NR | NR | NR | NR | 20.0 | NR |
| Wang | 2018 | 33.7 | 38.9 | 1.6 | 31.0 | 0.0 | 19.5 | 16.0 | 13.7 | NR | 28.5 | 36.8 | 72.5 | 34.6 | 65.4 |
| Wichter | 2004 | 43.0 | 81.7 | 32.0 | NR | 100.0 | 48.0 | NR | NR | 15.0 | 90.0 | NR | NR | 28.0 | NR |
| Zorzi | 2016 | 32.3 | 49.0 | NR | NR | NR | NR | NR | NR | NR | NR | 6.0 | NR | NR | NR |
| Aquaro | 2020 | 42.0 | 69.0 | NR | NR | 36.4 | 30.0 | NR | NR | 23.0 | NR | 56.0 | 39.0 | NR | NR |
| Cadrin-Tourigny | 2021 | 39.5 | 53.4 | NR | NR | 52.1 | 27.0 | 17.6 | NR | NR | 38.8 |  | 65.0 | 72.5 | 27.5 |
| Dominguez | 2020 | 39.1 | 59.7 | NR | NR | NR | NR | NR | NR | NR | NR | NR | NR | NR | NR |
| Feliu | 2020 | 48.6 | 67.6 | 47.9 | 21.6 | 42.5 | NR | NR | 31.1 | 28.2 | 14.9 | NR | 82.2 | NR | NR |
| Liang | 2020 | 38.2 | 81.3 | 9.9 | 34.5 | 14.4 | 65.1 | 22.2 | NR | 21.1 | NR | NR | NR | NR | NR |
| Lie | 2021 | 40.0 | 55.0 | 36.9 | 56.0 | NR | 14.0 | NR | NR | NR | NR | NR | 79.0 | 40.0 | NR |
| Saguner | 2014 | 42.9 | 67.0 | 14.0 | 63.0 | 70.0 | 39.0 | NR | 6.0 | NR | 57.0 | NR | NR | 29.0 | NR |
| Scheirlynck | 2020 | 47.0 | 51.0 | NR | NR | 43.0 | 7.0 | NR | 0.0 | 48.0 | NR | NR | 76.0 | NR | NR |
| Wozniak | 2021 | 39.2 | 73.9 | 41.9 | 40.3 | 100.0 | NR | 26.2 | NR | 32.3 | 72.3 | NR | NR | NR | NR |
| Bhonsale | 2011 | 31.9 | 46.0 | 14.0 | NR | 100.0 | NR | NR | 17.0 | 17.0 | NR | 49.0 | 57.0 | 76.0 | 24.0 |
| Link | 2014 | 40.0 | 60.0 | NR | NR | 100.0 | 55.0 | NR | NR | 19.4 | NR | 16.0 | NR | NR | NR |
| Oregon | 2017 | 33.6 | 52.0 | NR | 62.0 | 100.0 | NR | NR | NR | NR | NR | 37.0 | 60.0 | NR | NR |
| Platonov | 2019 | NR | 68.9 | 7.8 | NR | NR | NR | NR | 9.3 | NR | NR | NR | 51.5 | NR | NR |

AAD, anti-arrhythmic drug use; FHx, family history; ICD, implantable cardioverter-defibrillator; LVD, left ventricular dysfunction; NSVT, non-sustained ventricular tachycardia; RVD, right ventricular dysfunction; SCD, sudden cardiac death; SVT, sustained ventricular tachycardia; VT, ventricular tachycardia

## Table S4. Newcastle-Ottawa risk of bias estimation

| **Author** | **Year** | **Item 1** | **Item 2** | **Item 3** | **Item 4** | **Item 5** | **Item 6** | **Item 7** | **Item 8** | **Item 9** | **Score** | **RoB** |
| --- | --- | --- | --- | --- | --- | --- | --- | --- | --- | --- | --- | --- |
| Akdis | 2017 | 0 | 1 | 1 | 0 | 1 | 1 | 1 | 0 | 1 | 6 | Moderate |
| Blusztein | 2018 | 1 | 1 | 0 | 0 | 0 | 0 | 1 | 0 | 0 | 3 | High |
| Brun | 2016 | 0 | 0 | 1 | 1 | 0 | 1 | 1 | 1 | 1 | 6 | Moderate |
| Cadrin-Tourigny | 2019 | 1 | 1 | 1 | 1 | 1 | 1 | 1 | 1 | 1 | 9 | Low |
| Canpolat | 2013 | 0 | 1 | 1 | 0 | 1 | 1 | 1 | 1 | 1 | 7 | Moderate |
| Cappelletto | 2018 | 1 | 1 | 1 | 1 | 0 | 1 | 1 | 1 | 1 | 8 | Low |
| Chan | 2015 | 1 | 1 | 1 | 0 | 0 | 0 | 1 | 1 | 1 | 6 | Moderate |
| Chung | 2013 | 1 | 1 | 1 | 0 | 0 | 0 | 1 | 1 | 1 | 6 | Moderate |
| Chung | 2016 | 1 | 1 | 1 | 0 | 1 | 1 | 1 | 1 | 1 | 8 | Low |
| Corrado | 2003 | 1 | 0 | 1 | 1 | 1 | 0 | 1 | 1 | 1 | 7 | Moderate |
| Corrado | 2010 | 1 | 0 | 1 | 0 | 1 | 0 | 0 | 1 | 1 | 5 | Moderate |
| Denis | 2014 | 1 | 1 | 1 | 1 | 1 | 0 | 1 | 1 | 1 | 8 | Low |
| Gallo | 2016 | 1 | 1 | 0 | 1 | 0 | 1 | 0 | 1 | 1 | 6 | Moderate |
| Groeneweg | 2015 | 0 | 1 | 1 | 0 | 0 | 0 | 0 | 1 | 1 | 4 | Moderate |
| Hulot | 2004 | 1 | 1 | 1 | 1 | 0 | 0 | 1 | 1 | 1 | 7 | Moderate |
| Incardi | 2014 | 0 | 1 | 0 | 1 | 0 | 0 | 0 | 0 | 1 | 3 | High |
| James | 2013 | 1 | 1 | 1 | 1 | 0 | 0 | 1 | 1 | 1 | 7 | Moderate |
| Kikuchi | 2016 | 1 | 1 | 1 | 0 | 1 | 0 | 1 | 1 | 1 | 7 | Moderate |
| Kimura | 2018 | 0 | 0 | 1 | 0 | 1 | 0 | 1 | 1 | 1 | 5 | Moderate |
| Lemola | 2005 | 1 | 1 | 1 | 0 | 1 | 1 | 1 | 1 | 1 | 8 | Low |
| Li | 2012 | 0 | 1 | 0 | 0 | 0 | 0 | 1 | 1 | 1 | 4 | Moderate |
| Ma | 2009 | 0 | 0 | 0 | 0 | 0 | 0 | 1 | 1 | 1 | 3 | High |
| Maupain | 2018 | 1 | 1 | 0 | 1 | 0 | 1 | 1 | 1 | 1 | 7 | Low |
| Mazzanti | 2016 | 1 | 1 | 0 | 0 | 1 | 0 | 0 | 1 | 1 | 5 | Moderate |
| Migliore | 2013 | 1 | 1 | 1 | 0 | 1 | 1 | 1 | 1 | 1 | 8 | Low |
| Nava | 1988 | 0 | 0 | 1 | 0 | 0 | 0 | 1 | 0 | 0 | 2 | High |
| Peters | 1995 | 0 | 0 | 1 | 1 | 0 | 0 | 1 | 0 | 0 | 3 | High |
| Peters | 2012 | 0 | 1 | 1 | 1 | 0 | 1 | 0 | 1 | 1 | 6 | Moderate |
| Pinamonti | 2011 | 0 | 1 | 0 | 1 | 0 | 1 | 0 | 1 | 1 | 5 | Moderate |
| Protonotarios | 2015a | 1 | 1 | 1 | 0 | 1 | 0 | 1 | 1 | 1 | 7 | Moderate |
| Protonotarios | 2015b | 0 | 1 | 1 | 1 | 0 | 0 | 1 | 1 | 1 | 6 | Moderate |
| Roguin | 2004 | 0 | 1 | 1 | 0 | 0 | 0 | 1 | 1 | 1 | 5 | Moderate |
| Saguner | 2013 | 1 | 0 | 1 | 0 | 1 | 1 | 1 | 1 | 1 | 7 | Moderate |
| Saguner | 2014 | 1 | 1 | 1 | 1 | 0 | 1 | 0 | 1 | 1 | 7 | Moderate |
| Sen-Chowdhry | 2008 | 0 | 1 | 1 | 1 | 0 | 0 | 1 | 1 | 1 | 6 | Moderate |
| Turrini | 2001 | 1 | 1 | 1 | 1 | 0 | 1 | 1 | 0 | 0 | 6 | Moderate |
| Wang | 2018 | 1 | 1 | 1 | 1 | 1 | 1 | 1 | 1 | 1 | 9 | Low |
| Wichter | 2004 | 0 | 1 | 1 | 1 | 0 | 0 | 1 | 1 | 1 | 6 | Moderate |
| Zorzi | 2016 | 1 | 1 | 1 | 0 | 0 | 0 | 1 | 1 | 1 | 6 | Moderate |
| Aquaro | 2020 | 1 | 1 | 1 | 1 | 1 | 0 | 1 | 1 | 1 | 8 | Low |
| Cadrin-Tourigny | 2021 | 1 | 0 | 1 | 1 | 1 | 1 | 1 | 1 | 1 | 8 | Low |
| Dominguez | 2020 | 0 | 0 | 1 | 1 | 0 | 0 | 0 | 1 | 1 | 4 | Moderate |
| Feliu | 2020 | 1 | 1 | 1 | 0 | 1 | 0 | 1 | 1 | 1 | 7 | Moderate |
| Liang | 2020 | 1 | 1 | 1 | 1 | 1 | 0 | 1 | 0 | 1 | 7 | Moderate |
| Lie | 2021 | 1 | 1 | 1 | 0 | 1 | 0 | 1 | 1 | 1 | 7 | Moderate |
| Saguner | 2014 | 1 | 1 | 1 | 0 | 1 | 0 | 1 | 1 | 1 | 7 | Moderate |
| Scheirlynck | 2020 | 1 | 1 | 1 | 0 | 1 | 0 | 1 | 0 | 1 | 6 | Moderate |
| Wozniak | 2021 | 1 | 1 | 1 | 0 | 1 | 1 | 1 | 1 | 1 | 8 | Low |
| Bhonsale | 2011 | 0 | 1 | 1 | 1 | 0 | 1 | 0 | 1 | 1 | 6 | Moderate |
| Link | 2014 | 0 | 1 | 1 | 1 | 0 | 0 | 1 | 1 | 1 | 6 | Moderate |
| Oregon | 2017 | 0 | 1 | 1 | 1 | 1 | 1 | 1 | 1 | 1 | 8 | Low |
| Platonov | 2019 | 0 | 1 | 0 | 1 | 0 | 0 | 0 | 1 | 1 | 4 | High |

RoB, risk of bias

## Table S5. Summary of Annualised Incidence Rates of Appropriate Implantable-Cardioverter Defibrillator Therapy

| **Subgroup** | **Studies (*k*)** | **Participants** | | **Incidence rate**  **(95% CI)** | **Heterogeneity** | | ***Egger’s* test**  **(*P*-value)** |
| --- | --- | --- | --- | --- | --- | --- | --- |
|  |  | **No** | **Events** |  | ***I^2^*** | ***P*-value** |  |
| - Overall | 12 | 1,281 | 628 | 84.70 (64.18–105.22) | 36.9% | 0.096 | 0.050 |
| - Definite ARVC | 5 | 776 | 407 | 86.94 (51.98–121.89) | 63.3% | 0.028 | 0.140 |
| - **By pedigree** |  |  |  |  |  |  |  |
| - Probands only | 6 | 745 | 389 | 85.45 (59.00–111.90) | 31.9% | 0.197 | 0.637 |
| - Probands plus familial | 3 | 348 | 149 | 66.05 (27.66–104.45) | 38.4% | 0.197 | 0.100 |
| - **By Task Force Criteria** |  |  |  |  |  |  |  |
| - 2010 TFC | 6 | 813 | 432 | 74.28 (52.17–96.38) | 22.3% | 0.266 | 0.165 |
| - 1994 TFC | 4 | 406 | 178 | 104.82 (53.40–156.25) | 67.0% | 0.028 | 0.218 |
| - **By region** |  |  |  |  |  |  |  |
| - European cohorts | 8 | 584 | 282 | 67.39 (47.17–87.62) | 0.0% | 0.507 | 0.002 |
| - Trans-Atlantic cohorts | 3 | 589 | 298 | 88.71 (43.52–133.90) | 71.1% | 0.031 | 0.684 |

# Supplementary Figures

## Figure S1. Funnel plot of incidence rate of prospectively reported SCD in ICD-ve ARVC

## Figure S2. Bias-corrected incidence rate of prospectively reported SCD in ICD-ve ARVC

## Figure S3. Funnel plot of bias-corrected incidence of prospectively reported SCD in ICD-ve ARVC

## Figure S4. Pooled prevalence of retrospectively reported SCD or aborted SCD

##

## Figure S5. Univariate correlates of SCD

# Included Studies

1. Akdis D, Saguner AM, Shah K, Wei C, Medeiros-Domingo A, von Eckardstein A, Lüscher TF, Brunckhorst C, Chen HSV, Duru F. Sex hormones affect outcome in arrhythmogenic right ventricular cardiomyopathy/dysplasia: from a stem cell derived cardiomyocyte-based model to clinical biomarkers of disease outcome. Eur Heart J 2017;38(19):1498-1508.
2. Brun F, Groeneweg JA, Gear K, Sinagra G, van der Heijden J, Mestroni L, Hauer RN, Borgstrom M, Marcus FI, Hughes T. Risk Stratification in Arrhythmic Right Ventricular Cardiomyopathy Without Implantable Cardioverter-Defibrillators. JACC Clin Electrophysiol 2016;2(5):558-564.
3. Cadrin-Tourigny J, Bosman LP, Nozza A, Wang W, Tadros R, Bhonsale A, Bourfiss M, Fortier A, Lie ØH, Saguner AM, Svensson A, Andorin A, Tichnell C, Murray B, Zeppenfeld K, Van Den Berg MP, Asselbergs FW, Wilde AAM, Krahn AD, Talajic M, Rivard L, Chelko S, Zimmerman SL, Kamel IR, Crosson JE, Judge DP, Yap SC, Van Der Heijden JF, Tandri H, Jongbloed JDH, Guertin MC, Van Tintelen JP, Platonov PG, Duru F, Haugaa KH, Khairy P, Hauer RNW, Calkins H, Te Riele ASJM, James CA. A new prediction model for ventricular arrhythmias in arrhythmogenic right ventricular cardiomyopathy. European Heart Journal 2019;40(23):1850-1858.
4. Canpolat U, Kabakçi G, Aytemir K, Dural M, Sahiner L, Yorgun H, Sunman H, Bariş Kaya E, Tokgözoğlu L, Oto A. Fragmented QRS complex predicts the arrhythmic events in patients with arrhythmogenic right ventricular cardiomyopathy/dysplasia. J Cardiovasc Electrophysiol 2013;24(11):1260-6.
5. Cappelletto C, Stolfo D, De Luca A, Pinamonti B, Barbati G, Pivetta A, Gobbo M, Brun F, Merlo M, Sinagra G. Lifelong arrhythmic risk stratification in arrhythmogenic right ventricular cardiomyopathy: distribution of events and impact of periodical reassessment. Europace 2018;20(Fi1):f20-f29.
6. Chan CS, Lin YJ, Chang SL, Lo LW, Hu YF, Chao TF, Chung FP, Liao JN, Chen YJ, Chen SA. Early repolarization of surface ECG predicts fatal ventricular arrhythmias in patients with arrhythmogenic right ventricular dysplasia/cardiomyopathy and symptomatic ventricular arrhythmias. Int J Cardiol 2015;197:300-5.
7. Chung FP, Li HR, Chong E, Pan CH, Lin YJ, Chang SL, Lo LW, Hu YF, Tuan TC, Chao TF, Liao JN, Lin WY, Shaw KP, Chen SA. Seasonal variation in the frequency of sudden cardiac death and ventricular tachyarrhythmia in patients with arrhythmogenic right ventricular dysplasia/cardiomyopathy: the effect of meteorological factors. Heart Rhythm 2013;10(12):1859-66.
8. Chung FP, Lin YJ, Chong E, Chang SL, Lo LW, Hu YF, Tuan TC, Chao TF, Liao JN, Chen SA. The Application of Ambulatory Electrocardiographically-Based T-Wave Alternans in Patients with Arrhythmogenic Right Ventricular Dysplasia/Cardiomyopathy. Can J Cardiol 2016;32(11):1355.e15-1355.e22.
9. Corrado D, Basso C, Rizzoli G, Schiavon M, Thiene G. Does sports activity enhance the risk of sudden death in adolescents and young adults? J Am Coll Cardiol 2003;42(11):1959-63.
10. Corrado D, Calkins H, Link MS, Leoni L, Favale S, Bevilacqua M, Basso C, Ward D, Boriani G, Ricci R, Piccini JP, Dalal D, Santini M, Buja G, Iliceto S, Estes NA, 3rd, Wichter T, McKenna WJ, Thiene G, Marcus FI. Prophylactic implantable defibrillator in patients with arrhythmogenic right ventricular cardiomyopathy/dysplasia and no prior ventricular fibrillation or sustained ventricular tachycardia. Circulation 2010;122(12):1144-52.
11. James CA, Bhonsale A, Tichnell C, Murray B, Russell SD, Tandri H, Tedford RJ, Judge DP, Calkins H. Exercise increases age-related penetrance and arrhythmic risk in arrhythmogenic right ventricular dysplasia/cardiomyopathy-associated desmosomal mutation carriers. J Am Coll Cardiol 2013;62(14):1290-1297.
12. Kikuchi N, Yumino D, Shiga T, Suzuki A, Hagiwara N. Long-Term Prognostic Role of the Diagnostic Criteria for Arrhythmogenic Right Ventricular Cardiomyopathy/Dysplasia. JACC Clin Electrophysiol 2016;2(1):107-115.
13. Kimura Y, Takaki H, Inoue YY, Oguchi Y, Nagayama T, Nakashima T, Kawakami S, Nagase S, Noda T, Aiba T, Shimizu W, Kamakura S, Sugimachi M, Yasuda S, Shimokawa H, Kusano K. Isolated late activation detected by magnetocardiography predicts future lethal ventricular arrhythmic events in patients with arrhythmogenic right ventricular cardiomyopathy. Circulation Journal 2018;82(1):78-86.
14. Li CH, Lin YJ, Huang JL, Wu TJ, Cheng CC, Lin WS, Tuan TC, Chang SL, Lo LW, Hu YF, Chao TF, Chung FP, Tsai CF, Tsao HM, Chen SA. Long-term follow-up in patients with arrhythmogenic right ventricular cardiomyopathy. J Cardiovasc Electrophysiol 2012;23(7):750-6.
15. Liu T, Pursnani A, Sharma UC, Vorasettakarnkij Y, Verdini D, Deeprasertkul P, Lee AM, Lumish H, Sidhu MS, Medina H, Danik S, Abbara S, Holmvang G, Hoffmann U, Ghoshhajra BB. Effect of the 2010 task force criteria on reclassification of cardiovascular magnetic resonance criteria for arrhythmogenic right ventricular cardiomyopathy. J Cardiovasc Magn Reson 2014;16(1):47.
16. Maupain C, Badenco N, Pousset F, Waintraub X, Duthoit G, Chastre T, Himbert C, Hébert JL, Frank R, Hidden-Lucet F, Gandjbakhch E. Risk Stratification in Arrhythmogenic Right Ventricular Cardiomyopathy/Dysplasia Without an Implantable Cardioverter-Defibrillator. JACC Clin Electrophysiol 2018;4(6):757-768.
17. Mazzanti A, Ng K, Faragli A, Maragna R, Chiodaroli E, Orphanou N, Monteforte N, Memmi M, Gambelli P, Novelli V, Bloise R, Catalano O, Moro G, Tibollo V, Morini M, Bellazzi R, Napolitano C, Bagnardi V, Priori SG. Arrhythmogenic Right Ventricular Cardiomyopathy: Clinical Course and Predictors of Arrhythmic Risk. J Am Coll Cardiol 2016;68(23):2540-2550.
18. Nava A, Thiene G, Canciani B, Scognamiglio R, Daliento L, Buja G, Martini B, Stritoni P, Fasoli G. Familial occurrence of right ventricular dysplasia: a study involving nine families. J Am Coll Cardiol 1988;12(5):1222-8.
19. Peters S, Reil GH. Risk factors of cardiac arrest in arrhythmogenic right ventricular dysplasia. Eur Heart J 1995;16(1):77-80.
20. Peters S, Truemmel M, Koehler B. Prognostic value of QRS fragmentation in patients with arrhythmogenic right ventricular cardiomyopathy/dysplasia. J Cardiovasc Med (Hagerstown) 2012;13(5):295-8.
21. Pinamonti B, Dragos AM, Pyxaras SA, Merlo M, Pivetta A, Barbati G, Di Lenarda A, Morgera T, Mestroni L, Sinagra G. Prognostic predictors in arrhythmogenic right ventricular cardiomyopathy: results from a 10-year registry. Eur Heart J 2011;32(9):1105-13.
22. Protonotarios A, Anastasakis A, Panagiotakos DB, Ritsatos C, Vlagouli V, Syrris P, Tousoulis D, Antoniades L, McKenna WJ, Tsatsopoulou A. First arrhythmic event-associated clinical disease profile in arrhythmogenic cardiomyopathy associated desmosomal mutation carriers. European Heart Journal 2015;36:453.
23. Protonotarios A, Anastasakis A, Tsatsopoulou A, Antoniades L, Prappa E, Syrris P, Tousoulis D, Mc KW, Protonotarios N. Clinical Significance of Epsilon Waves in Arrhythmogenic Cardiomyopathy. J Cardiovasc Electrophysiol 2015;26(11):1204-1210.
24. Roguin A, Bomma CS, Nasir K, Tandri H, Tichnell C, James C, Rutberg J, Crosson J, Spevak PJ, Berger RD, Halperin HR, Calkins H. Implantable cardioverter-defibrillators in patients with arrhythmogenic right ventricular dysplasia/cardiomyopathy. J Am Coll Cardiol 2004;43(10):1843-52.
25. Saguner AM, Ganahl S, Baldinger SH, Kraus A, Medeiros-Domingo A, Nordbeck S, Saguner AR, Mueller-Burri AS, Haegeli LM, Wolber T, Steffel J, Krasniqi N, Delacrétaz E, Lüscher TF, Held L, Brunckhorst CB, Duru F. Usefulness of electrocardiographic parameters for risk prediction in arrhythmogenic right ventricular dysplasia. Am J Cardiol 2014;113(10):1728-34.
26. Sen-Chowdhry S, Syrris P, Prasad SK, Hughes SE, Merrifield R, Ward D, Pennell DJ, McKenna WJ. Left-dominant arrhythmogenic cardiomyopathy: an under-recognized clinical entity. J Am Coll Cardiol 2008;52(25):2175-87.
27. Wang W, Cadrin-Tourigny J, Bhonsale A, Tichnell C, Murray B, Monfredi O, Chrispin J, Crosson J, Tandri H, James CA, Calkins H. Arrhythmic outcome of arrhythmogenic right ventricular cardiomyopathy patients without implantable defibrillators. J Cardiovasc Electrophysiol 2018;29(10):1396-1402.
28. Wichter T, Paul M, Wollmann C, Acil T, Gerdes P, Ashraf O, Tjan TDT, Soeparwata R, Block M, Borggrefe M, Scheld HH, Breithardt G, Bocker D. Implantable cardioverter/defibrillator therapy in arrhythmogenic right ventricular cardiomyopathy - Single-center experience of long-term follow-up and complications in 60 patients. Circulation 2004;109(12):1503-1508.
29. Zorzi A, Rigato I, Pilichou K, Perazzolo Marra M, Migliore F, Mazzotti E, Gregori D, Thiene G, Daliento L, Iliceto S, Rampazzo A, Basso C, Bauce B, Corrado D. Phenotypic expression is a prerequisite for malignant arrhythmic events and sudden cardiac death in arrhythmogenic right ventricular cardiomyopathy. Europace 2016;18(7):1086-94.
30. Blusztein DI, Zentner D, Thompson T, Jayadeva P, Liang D, Wang R, Winship I, James PA, Trainer AH, Kalman JM, Vohra J. Arrhythmogenic Right Ventricular Cardiomyopathy: A Review of Living and Deceased Probands. Heart Lung Circ 2019;28(7):1034-1041.
31. Gallo C, Blandino A, Giustetto C, Anselmino M, Castagno D, Richiardi E, Gaita F. Arrhythmogenic right ventricular cardiomyopathy: ECG progression over time and correlation with long-term follow-up. J Cardiovasc Med (Hagerstown) 2016;17(6):418-24.
32. Groeneweg JA, Bhonsale A, James CA, te Riele AS, Dooijes D, Tichnell C, Murray B, Wiesfeld AC, Sawant AC, Kassamali B, Atsma DE, Volders PG, de Groot NM, de Boer K, Zimmerman SL, Kamel IR, van der Heijden JF, Russell SD, Jan Cramer M, Tedford RJ, Doevendans PA, van Veen TA, Tandri H, Wilde AA, Judge DP, van Tintelen JP, Hauer RN, Calkins H. Clinical Presentation, Long-Term Follow-Up, and Outcomes of 1001 Arrhythmogenic Right Ventricular Dysplasia/Cardiomyopathy Patients and Family Members. Circ Cardiovasc Genet 2015;8(3):437-46.
33. Hulot JS, Jouven X, Empana JP, Frank R, Fontaine G. Natural history and risk stratification of arrhythmogenic right ventricular dysplasia/cardiomyopathy. Circulation 2004;110(14):1879-84.
34. Inciardi RM, Maresi E, Coppola G, Rotolo A, Clemenza F, Giordano U, Lombardo E, Schicchi R, Torcivia R, Arrotti S, Iacona R, Minacapelli AA, Assennato P, Novo S. Anatomical features and clinical correlations in Caucasian patients with definite arrhythmogenic right ventricular dysplasia/cardiomyopathy. Minerva Cardioangiol 2014;62(5):369-78.
35. Aquaro GD, De Luca A, Cappelletto C, Raimondi F, Bianco F, Botto N, Barison A, Romani S, Lesizza P, Fabris E, Todiere G, Grigoratos C, Pingitore A, Stolfo D, Dal Ferro M, Merlo M, Di Bella G, Sinagra G. Comparison of different prediction models for the indication of implanted cardioverter defibrillator in patients with arrhythmogenic right ventricular cardiomyopathy. Esc Heart Failure 2020;7(6):4080-4088.
36. Cadrin-Tourigny J, Bosman LP, Wang W, Tadros R, Bhonsale A, Bourfiss M, Lie Ø, Saguner AM, Svensson A, Andorin A, Tichnell C, Murray B, Zeppenfeld K, van den Berg MP, Asselbergs FW, Wilde AAM, Krahn AD, Talajic M, Rivard L, Chelko S, Zimmerman SL, Kamel IR, Crosson JE, Judge DP, Yap SC, Van der Heijden JF, Tandri H, Jongbloed JDH, van Tintelen JP, Platonov PG, Duru F, Haugaa KH, Khairy P, Hauer RNW, Calkins H, Te Riele ASJM, James CA. Sudden Cardiac Death Prediction in Arrhythmogenic Right Ventricular Cardiomyopathy: A Multinational Collaboration. Circulation. Arrhythmia and electrophysiology 2021;14(1):e008509.
37. Dominguez F, Zorio E, Jimenez-Jaimez J, Salguero-Bodes R, Zwart R, Gonzalez-Lopez E, Molina P, Bermudez-Jimenez F, Delgado JF, Braza-Boils A, Bornstein B, Toquero J, Segovia J, Van Tintelen JP, Lara-Pezzi E, Garcia-Pavia P. Clinical characteristics and determinants of the phenotype in TMEM43 arrhythmogenic right ventricular cardiomyopathy type 5. Heart Rhythm 2020;17(6):945-954.
38. Feliu E, Moscicki R, Carrillo L, Garcia-Fernandez A, Martinez JGM, Ruiz-Nodar JM. Importance of cardiac magnetic resonance findings in the diagnosis of left dominant arrythmogenic cardiomyopathy. Revista Espanola De Cardiologia 2020;73(11):885-892.
39. Liang E, Wu L, Fan S, Hu F, Zheng L, Liu S, Fan X, Chen G, Ding L, Niu G, Yao Y. Catheter ablation of arrhythmogenic right ventricular cardiomyopathy ventricular tachycardia: 18-year experience in 284 patients. Europace 2020;22(5):806-812.
40. Scheirlynck E, Chivulescu M, Lie OH, Motoc A, Koulalis J, de Asmundis C, Sieira J, Chierchia GB, Brugada P, Cosyns B, Edvardsen T, Droogmans S, Haugaa KH. Worse Prognosis in Brugada Syndrome Patients With Arrhythmogenic Cardiomyopathy Features. Jacc-Clinical Electrophysiology 2020;6(11):1353-1363.
41. Woźniak O, Borowiec K, Konka M, Cicha-Mikołajczyk A, Przybylski A, Szumowski Ł, Hoffman P, Poślednik K, Biernacka EK. Implantable cardiac defibrillator events in patients with arrhythmogenic right ventricular cardiomyopathy. Heart 2021.
42. Denis A, Sacher F, Derval N, Lim HS, Cochet H, Shah AJ, Daly M, Pillois X, Ramoul K, Komatsu Y, Zemmoura A, Amraoui S, Ritter P, Ploux S, Bordachar P, Hocini M, Jaïs P, Haïssaguerre M. Diagnostic value of isoproterenol testing in arrhythmogenic right ventricular cardiomyopathy. Circ Arrhythm Electrophysiol 2014;7(4):590-7.
43. Lemola K, Brunckhorst C, Helfenstein U, Oechslin E, Jenni R, Duru F. Predictors of adverse outcome in patients with arrhythmogenic right ventricular dysplasia/cardiomyopathy: long term experience of a tertiary care centre. Heart 2005;91(9):1167-72.
44. Ma KJ, Li N, Wang HT, Chu JM, Fang PH, Yao Y, Ma J, Hua W, Zhang S, Wang FZ, Li Z, Pu JL. Clinical study of 39 Chinese patients with arrhythmogenic right ventricular dysplasia/cardiomyopathy. Chinese Medical Journal 2009;122(10):1133-1138.
45. Migliore F, Zorzi A, Silvano M, Bevilacqua M, Leoni L, Marra MP, Elmaghawry M, Brugnaro L, Dal Lin C, Bauce B, Rigato I, Tarantini G, Basso C, Buja G, Thiene G, Iliceto S, Corrado D. Prognostic Value of Endocardial Voltage Mapping in Patients With Arrhythmogenic Right Ventricular Cardiomyopathy/Dysplasia. Circulation-Arrhythmia and Electrophysiology 2013;6(1):167-176.
46. Turrini P, Corrado D, Basso C, Nava A, Bauce B, Thiene G. Dispersion of ventricular depolarization-repolarization - A noninvasive marker for risk stratification in arrhythmogenic right ventricular cardiomyopathy. Circulation 2001;103(25):3075-3080.
47. Orgeron GM, James CA, Te Riele A, Tichnell C, Murray B, Bhonsale A, Kamel IR, Zimmerman SL, Judge DP, Crosson J, Tandri H, Calkins H. Implantable Cardioverter-Defibrillator Therapy in Arrhythmogenic Right Ventricular Dysplasia/Cardiomyopathy: Predictors of Appropriate Therapy, Outcomes, and Complications. J Am Heart Assoc 2017;6(6).
48. Platonov PG, Haugaa KH, Bundgaard H, Svensson A, Gilljam T, Hansen J, Madsen T, Host AG, Carlson J, Lie OH, Jensen MK, Edvardsen T, Jensen HK, Svendsen JH. Primary Prevention of Sudden Cardiac Death With Implantable Cardioverter-Defibrillator Therapy in Patients With Arrhythmogenic Right Ventricular Cardiomyopathy. American Journal of Cardiology 2019;123(7):1156-1162.
49. Bhonsale A, James CA, Tichnell C, Murray B, Gagarin D, Philips B, Dalal D, Tedford R, Russell SD, Abraham T, Tandri H, Judge DP, Calkins H. Incidence and predictors of implantable cardioverter-defibrillator therapy in patients with arrhythmogenic right ventricular dysplasia/cardiomyopathy undergoing implantable cardioverter-defibrillator implantation for primary prevention. J Am Coll Cardiol 2011;58(14):1485-96.
50. Saguner AM, Medeiros-Domingo A, Schwyzer MA, On CJ, Haegeli LM, Wolber T, Hürlimann D, Steffel J, Krasniqi N, Rüeger S, Held L, Lüscher TF, Brunckhorst C, Duru F. Usefulness of inducible ventricular tachycardia to predict long-term adverse outcomes in arrhythmogenic right ventricular cardiomyopathy. Am J Cardiol 2013;111(2):250-7.
51. Lie Ø H, Chivulescu M, Rootwelt-Norberg C, Ribe M, Bogsrud MP, Lyseggen E, Beitnes JO, Almaas V, Haugaa KH. Left Ventricular Dysfunction in Arrhythmogenic Cardiomyopathy: Association With Exercise Exposure, Genetic Basis, and Prognosis. J Am Heart Assoc 2021;10(8):e018680.
52. Link MS, Laidlaw D, Polonsky B, Zareba W, McNitt S, Gear K, Marcus F, Estes Iii NAM. Ventricular arrhythmias in the North American multidisciplinary study of ARVC: Predictors, characteristics, and treatment. Journal of the American College of Cardiology 2014;64(2):119-125.
